# Identification and Functional Analysis of a Novel von Willebrand Factor Mutation in a Family with Type 2A von Willebrand Disease

**DOI:** 10.1371/journal.pone.0033263

**Published:** 2012-03-27

**Authors:** Jing Dong, Xiaojuan Zhao, Sensen Shi, Zhenni Ma, Meng Liu, Qingyu Wu, Changgeng Ruan, Ningzheng Dong

**Affiliations:** 1 Jiangsu Institute of Hematology, Soochow University, First Affiliated Hospital, Suzhou, China; 2 Cyrus Tang Hematology Center, Soochow University, Suzhou, China; 3 Molecular Cardiology, Nephrology and Hypertension, Lerner Research Institute, Cleveland Clinic, Cleveland, Ohio, United States of America; 4 Key Lab of Thrombosis and Hemostasis of Ministry of Health, Jiangsu Institute of Hematology, Soochow University, First Affiliated Hospital, Suzhou, China; Leiden University Medical Center, The Netherlands

## Abstract

von Willebrand factor (VWF) is essential for normal hemostasis. VWF gene mutations cause the hemorrhagic von Willebrand disease (VWD). In this study, a 9-year-old boy was diagnosed as type 2A VWD, based on a history of abnormal bleeding, low plasma VWF antigen and activity, low plasma factor VIII activity, and lack of plasma high-molecular-weight (HMW) VWF multimers. Sequencing analysis detected a 6-bp deletion in exon 28 of his VWF gene, which created a mutant lacking D1529V1530 residues in VWF A2 domain. This mutation also existed in his family members with abnormal bleedings but not in >60 normal controls. In transfected HEK293 cells, recombinant VWF ΔD1529V1530 protein had markedly reduced levels in the conditioned medium (42±4% of wild-type (WT) VWF, *p*<0.01). The mutant VWF in the medium had less HMW multimers. In contrast, the intracellular levels of the mutant VWF in the transfected cells were significantly higher than that of WT (174±29%, *p*<0.05), indicating intracellular retention of the mutant VWF. In co-transfection experiments, the mutant reduced WT VWF secretion from the cells. By immunofluorescence staining, the retention of the mutant VWF was identified within the endoplasmic reticulum (ER). Together, we identified a unique VWF mutation responsible for the bleeding phenotype in a patient family with type 2A VWD. The mutation impaired VWF trafficking through the ER, thereby preventing VWF secretion from the cells. Our results illustrate the diversity of VWF gene mutations, which contributes to the wide spectrum of VWD.

## Introduction

von Willebrand factor (VWF) is a multimeric glycoprotein that plays an important role in hemostasis [1], [2]. VWF mediates platelet adhesion at damaged vascular sites and protects plasma factor VIII (FVIII) from proteolytic degradation. In vascular endothelial cells and megakaryocytes, VWF is synthesized as a 2813-amino acid processor consisting of multiple domains in the order of D1-D2-D′-D3-A1-A2-A3-D4-B1-B2-B3-C1-C2-CK. After removal of the signal peptide, VWF monomers in the endoplasmic reticulum (ER) form dimers by connecting their C-termini through disulfide bonds. VWF dimers then translocate to the Golgi to form high-molecular-weight (HMW) multimers by linking cysteine residues at their N-termini. The assembled VWF is either constitutively secreted or stored in Weibel-Palade bodies of endothelial cells or α-granules of platelets. The HMW multimers are critical for VWF to mediate platelet adhesion under high shear flow conditions [1], [2].

von Willebrand disease (VWD) is the most common inherited bleeding disorder caused by mutations in the VWF gene [3], [4], [5], [6], [7], [8], [9], [10], [11], [12], [13], [14], [15], [16], [17], [18], which result in quantitative and/or qualitative defects in VWF [2], [19]. VWD is classified into three types: types 1 and 3 refer to different degrees of quantitative VWF deficiencies, whereas type 2 refers to all qualitative VWF deficiencies. Type 2 VWD is subdivided into types 2A, 2B, 2M, and 2N. Type 2A refers to qualitative variants with decreased platelet adhesion activity and a selective loss of HMW VWF multimers [19]. Identification of VWF gene mutations in VWD subtypes has helped to understand the molecular basis underlying VWD and the structure-function relationship of VWF.

In this study, we diagnosed a patient family with type 2A VWD. DNA sequence analysis of the VWF gene in the patients identified a novel 6-base pair (bp) deletion in exon 28, which encodes the C-terminal half of VWF A2 domain. In functional studies, the mutation did not prevent VWF expression in transfected human embryonic kidney (HEK)293 cells but markedly impaired VWF secretion from the cells. The results help to explain the findings of low plasma VWF levels but normal platelet VWF levels in the patients and support the importance of A2 domain in VWF biosynthesis and secretion.

## Results

### Diagnosis of a patient family with type 2A VWD

**Figure 1:**
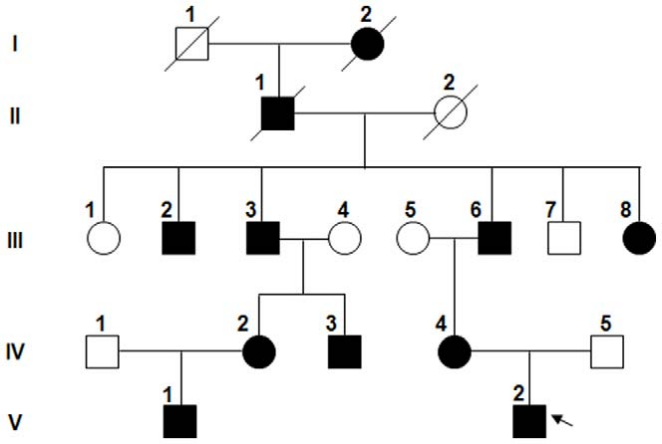
Pedigree of the family with bleeding disorder. Squares and circles indicate males and females, respectively, and arrow indicates the proband. Black color denotes affected individuals. A slash through the symbol indicates decreased individuals.

The proband was a 9-year-old boy, whose family pedigree is shown in Fig. 1. He suffered from frequent bruise, epistaxis, prolonged bleedings from small wounds, and delayed wound healing. His mother also had a history of epistaxis, menorrhagia and anemia. His father did not experience any abnormal bleedings. Several members in the extended family had bleeding tendencies. The results of laboratory tests from the proband and some of his family members are shown in Table 1, which indicated normal clotting times but low plasma VWF antigen and activity in the proband (V-2), and the family members III-3, IV-2, IV-3 and IV-4.

**Table 1:** Main laboratory findings of the patient and family members.

	V-2	III-3	IV-2	IV-3	IV-4	IV-5	Normal Range
TT, sec	16.7	16.7	15.6	18.6	18.1	17.1	14–21
APTT, sec	58.1	45.5	45.8	45.2	46.1	36.6	28–40
PT, sec	14.2	14.0	13.8	13.4	14.1	13.0	11–14.5
RIPA, %	28.5	N/A	N/A	N/A	3.0	85.0	50–150
VWF:Ag, U/dL	20.5	21.6	30.4	19.5	21.7	95.2	50–150
VWF:Rco, U/dL	6.2	5.0	8.4	7.6	5.0	94.5	50–150
FVIII:C, U/dL	25.0	28.0	50.0	29.0	N/A	79.0	50–150

TT, thrombin time; APTT, activated partial thromboplastin time; PT, prothrombin time; RIPA, ristocetin-induced platelet agglutination; VWF:Ag, VWF antigen; VWF:RCo, VWF ristocetin cofactor activity; FVIII:C, FVIII coagulant activity; N/A, not available.

**Figure 2:**
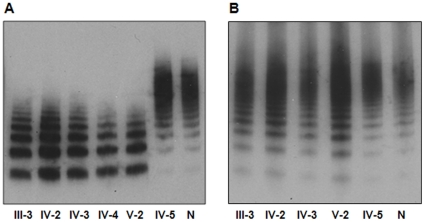
Analysis of plasma and platelet VWF multimers. Plasma (A) and platelet (B) VWF multimers were assessed by 1.6% SDS-agarose gel electrophoresis and Western blotting. Normal plasma samples were diluted 1∶20 and patient plasma samples were diluted 1∶5. Samples from family members and a normal control (N) were indicated. Platelet samples were not available from IV-4.

We analyzed VWF multimers in plasma and platelets from these individuals. Plasma VWF from the proband, III-3, IV-2, IV-3 and IV-4 lacked high or intermediate MW multimers, whereas VWF from the proband's father (IV-5) had a similar multimer pattern to that of a normal control (Fig. 2A). In contrast, platelet VWF from these individuals all had HMW multimers that were indistinguishable from those of normal controls (Fig. 2B). Based on these data and the results of laboratory tests, including low plasma VWF Ag and activities (Table 1), the proband and 4 additional family members (III-3, IV-2, IV-3 and IV-4) were diagnosed for type 2A VWD.

### Identification of a VWF gene mutation

**Figure 3:**
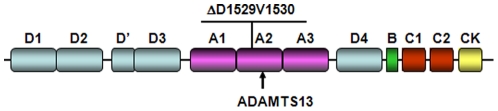
Identification of a VWF gene mutation. Sequence analysis of the VWF gene in the proband detected a 6-bp nucleotides deletion in exon 28. The mutation caused D1529V1530 deletion (ΔD1529V1530) in VWF A2 domain. The ADAMTS13 cleavage site is indicated by an arrow.

To understand the genetic basis underlying the VWD in this family, we isolated genomic DNA from the proband and sequenced all 52 exons in his VWF gene. We found overlapping peaks in the sequencing chromatogram of exon 28 (data not shown), suggesting a heterozygous mutation. To verify this VWF gene mutation, we separated DNA fragments from the patient's two alleles by PCR amplification and cloning of the DNA fragments into pMD18-T vector. Sequencing analysis of the resulting plasmids showed that the patient had one normal VWF allele and a mutant allele with a 6-bp deletion in exon 28, which encoded amino acids D1529 and V1530 in VWF A2 domain (Fig. 3). The deletion kept the remaining coding sequence in-frame. We did not find any additional insertion, deletion or non-synonymous point mutations in his VWF gene. This 6-bp deletion mutation also existed in his mother (IV-4) and three other family members (III-3, IV-2 and IV-3) with bleeding symptoms but not in his father (IV-5) and more than 60 unrelated normal controls, indicating that this mutation is responsible for the VWD in this family.

### Analysis of recombinant mutant VWF

We constructed a plasmid expressing mutant VWF with D1529V1530 deletion (ΔD1529V1530) and transfected the plasmid into HEK293 cells. Recombinant VWF in the condition medium and cell lysates was quantified by ELISA (Table 2). Compared with control recombinant wild-type (WT) VWF in similarly transfected cells, VWF ΔD1529V1530 mutant had a higher intracellular level (174±29%, *p*<0.05). The level of the mutant VWF in the conditioned medium, however, was markedly reduced compared to that of WT control (42±4%, *p*<0.01).

**Table 2:** Expression of WT and mutant VWF in transfected HEK293 cells.

Plasmid	Cell Lysate	Conditioned Medium		
	VWF:Ag (%)	VWF:Ag (%)	VWF:CB (%)	VWF:CB/VWF:Ag
WT	100±28	100±12	100±12	1.0
Mutant	174±29*	42±4**	16±4**	0.38
Mutant/WT	121±52	61±16*	21±16**	0.34
WT (1/2)	58±21	68±7*	32±7**	0.47

VWF:Ag, VWF antigen; VWF:CB, VWF collagen-binding activity; WT, wild-type VWF; WT (1/2), one-half amount of WT plasmid; Data represent mean ± SD,

** *p*<0.01 *vs.* WT,

* *p*<0.05 *vs.* WT. Data were from three independent experiments.

To test the hypothesis that the mutant VWF allele may interfere with the expression of the normal VWF allele in the patients, we mixed equal amounts of plasmids for WT and ΔD1529V1530 mutant VWF and transfected them into HEK293 cells. ELISA analysis of cell lysates showed higher levels of recombinant VWF expression in HEK293 cells transfected with both WT and ΔD1529V1530 mutant plasmids (Table 2). The levels in the conditioned medium from these cells, however, were significantly lower than that from the cells transfected with WT plasmid alone (61±16% of WT, *p*<0.05). The reduced levels were similar to that from the cells transfected with one-half amount of WT plasmid (Table 2). The results indicate that ΔD1529V1530 mutant impaired WT VWF secretion from the transfected cells.

**Figure 4:**
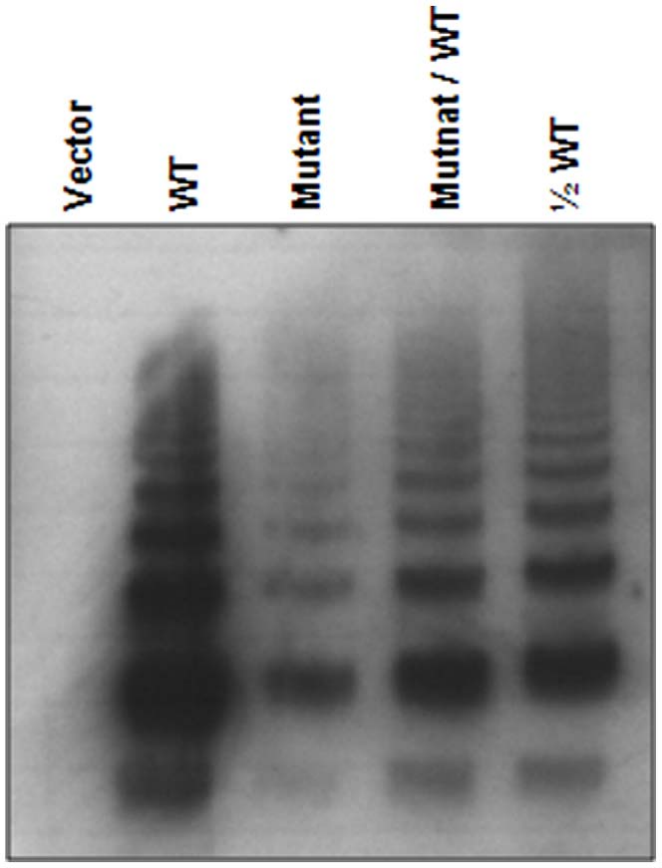
Multimer analysis of recombinant VWF expressed in HEK293 cells. Multimeric analysis of recombinant VWF was performed with the conditioned medium from HEK293 cells transfected with plasmids for WT and the mutant, individually or in combination. Samples from the vector-transfected cells were used as a negative control.

We then analyzed the multimer pattern of recombinant WT and mutant VWF from the transfected cells. As shown in Fig. 4, recombinant WT VWF from the conditioned medium had HMW multimers. In contrast, VWF ΔD1529V1530 mutant from similarly transfected cells had lower expression levels and less HMW multimers. In the conditioned media from the cells transfected with both the mutant and WT plasmids or one-half amount of WT plasmid alone, VWF protein expression levels were lower but HMW multimers were clearly present (Fig. 4).

**Figure 5:**
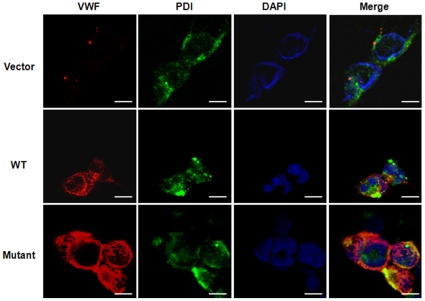
Detection of WT and mutant VWF in transfected HEK293 cells by immunostaining. HEK293 cells were transfected with a control vector or plasmids expressing WT or mutant VWF. The cells were stained for VWF (red), PDI (green) or DAPI (nucleus, blue). In the right column panels, merged pictures of green, red and blue channels are shown. The images were obtained with a confocal microscope. Scale bars: 10 µm.

We next did immunofluorescence staining to localize WT and mutant VWF proteins inside the cells by co-staining with protein disulfide isomerase (PDI), an ER protein marker. As shown in Fig. 5 (middle row panels), recombinant WT VWF was detected in transfected HEK293 cells. The protein was present around the nucleus and in the cytoplasm where the staining was granule-like. There were limited overlaps between VWF and PDI expression. In similarly transfected HEK293 cells, the mutant VWF was detected mostly around the nucleus and the expression pattern overlapped with that of PDI (Fig. 5, lower panels). The results indicate that a significant portion of the mutant VWF molecules was retained in the ER in these cells.

## Discussion

In this study, we report a patient family with type 2A VWD. The diagnosis was based on the VWD classification [9], [19], which includes a history of moderate bleeding, low levels of plasma VWF:Ag, reduced VWF:CB and VWF:Rco activities, decreased FVIII coagulant activity, and lack of high and intermediate MW VWF multimers. Sequencing analysis of the VWF gene exons of the proband identified a 6-bp deletion in exon 28. This mutation has never been reported in the literature and is predicted to create a mutant VWF that lacks residues D1529V1530 in A2 domain. This novel mutation was found in 4 additional family members, who had similar clinical symptoms and laboratory findings, but not in >60 normal controls.

We expressed and characterized VWF ΔD1529V1530 mutant in transfected HEK293 cells. Unlike the previously reported delR1569 mutation in VWF A2 domain, which greatly reduced VWF expression [20], the deletion of D1529V1530 did not prevent VWF expression in HEK293 cells, as indicated by higher levels of VWF protein in cell lysate (Table 2). The secretion of this mutant from the cells, however, was markedly impaired, as indicated by low levels of mutant VWF in the conditioned medium (Table 2). The mutant also decreased the levels of recombinant WT VWF in the conditioned medium when they were co-expressed, exhibiting a dominant-negative effect on VWF secretion (Table 2). These data were consistent with the findings of low plasma VWF levels but normal platelet VWF levels in these patients. Similar findings of normal cellular expression but reduced secretion were reported in other type 2A VWD patients who had mutations in VWF A2 [20], [21], D3 or CK domains [22]. The dominant-negative effect of ΔD1529V1530 mutant on WT VWF secretion in transfected cells may also explain the autosomal dominant inheritance observed in this patient family.

In patients with type 2A VWD, mutations in VWF A1 and A2 domains often resulted in the loss of HMW, and sometimes intermediate MW, multimers [7], [23]. It is proposed that type 2A VWD may be divided into groups I and II, based on different molecular defects [7], [11], [21], [24]. In group I, mutations impair the intracellular transport, storage or secretion of VWF, especially HMW multimers. The reported VWF mutations that belong to this group include L1503R [11], G1505R, S1506L [21], L1540P [11], [25] and V1607D [21]. In group II, mutations do not alter VWF protein synthesis, intracellular transport or secretion, but render the protein more susceptible to ADAMTS13, which cleaves VWF at Y1605-M1606 in A2 domain [7], [24], [26] (Fig. 3). These data indicate that mutations in A2 domain, depending on their specific positions and the nature of amino acid changes, may have different effects on the intracellular trafficking and posttranslational degradation of VWF.

Unlike most point mutations reported in type 2A VWD that alter single amino acids, the mutation we found in this study deleted two amino acids in A2 domain while the remaining VWF sequence was kept in-frame. Mechanistically, the type 2A VWD caused by this deletion mutation belongs to group I because the mutation impaired VWF secretion. As shown by ELISA and multimer analysis (Table 2 and Fig. 4), mutant VWF in the conditioned medium was at low levels and had less HMW multimers, consistent with low levels of plasma VWF that also lacked HMW mulitmers in these patients (Fig. 2A). We showed that co-expression of WT VWF did not significantly improve the secretion of the mutant in transfected cells (Table 2). In this regard, this mutant VWF differed from some of quantitative VWF mutants in D3 and CK domains, which also had reduced secretion in transfected HEK293 cells. The defective secretion of these quantitative VWF mutants, however, was attenuated if WT VWF was co-expressed, resulting in normal amounts of HMW multimers in the conditioned medium [27], [28]. These data indicate that ΔD1529V1530 mutant may interact with WT VWF and impair the intracellular trafficking and secretion of WT VWF. In the patients, however, it is unlikely that the mutation completely blocked the VWF intracellular trafficking since HMW VWF multimers were detected in the patients' platelets (Fig. 2A). The data suggest that ΔD1529V1530 mutation may impair both the intracellular trafficking and secretion of VWF in the patients.

In summary, we identified a novel deletion mutation in VWF A2 domain in a family of patients with type 2A VWD. In functional studies, we showed that the mutation did not prevent VWF expression but markedly impaired VWF secretion in transfected HEK293 cells. Our results help to determine the genetic basis of the bleeding phenotype in this patient family and provide new data to illustrate the diversity of VWF gene mutations that contributes to the wide spectrum of VWD.

## Materials and Methods

### Blood samples and laboratory tests

This study was approved by the Ethics Committees of the First Affiliated Hospital of Soochow University and all participants gave informed consent. Blood was collected in tubes containing 3.2% of sodium citrate [1∶10 (v/v)] as an anticoagulant. Plasma was prepared by centrifugation of blood samples at 12, 000× g for 5 min, aliquoted and stored at −80°C until use. Platelet-rich plasma (PRP) and platelet-poor plasma (PPP) were prepared by centrifugation of blood samples at 100× g for 10 min and 800× g for 15 min, respectively.

VWF antigen (VWF:Ag) and VWF ristocetin cofactor activity (VWF:Rco) were measured by ELISA [29]. VWF collagen-binding activity (VWF:CB) was measured using an ELISA-based assay with human type III collagen (Sigma, St Louis, MO, USA) [30]. Ristocetin-induced platelet aggregation (RIPA) was done on a platelet aggregometer (Chronolog) in the presence of 1.25 mg/mL ristocetin (Sigma). Plasma FVIII activity was measured by a one-stage method [31].

### VWF multimer analysis

Analysis of VWF multimers in plasma and platelets from patients or in the conditioned medium from transfected cells expressing recombinant VWF was performed using 1.6% SDS-agarose gel electrophoresis, as previously described [10], [32]. VWF multimers in the gel were transferred to a nitrocellulose membrane and detected by an HRP-conjugated rabbit anti-human VWF antibody (Dako, Glostrup, Denmark).

### DNA extraction and sequencing

Genomic DNA was extracted from peripheral blood leukocytes using the QIAamp Blood Kit (Qiagen), according to the manufacturer's instruction. DNA sequences from all 52 exons and intron–exon boundaries of the VWF gene were amplified by PCR using oligonucleotide primers that were listed publicly http://www.euvwd.group.shef.ac.uk/sequence.htm. PCR products were sequenced directly by a DNA analyzer (Applied Biosystems 310). To verify possible heterozygous deletions or insertions in patients, amplified DNA fragments with candidate mutations were cloned into pMD18-T vector (Takara), and inserts from the resulting plasmids were sequenced. The new sequence data has been deposited in GenBank (accession number: BankIt1476860 Seq1 JN625247).

### Plasmid construction

The plasmid pSVHVWF containing the full-length human VWF cDNA was kindly provided by Evan Sadler (Washington University School of Medicine, St Louis, USA) [33]. A PCR-based method was used to construct expression vectors encoding VWF mutants using the full-length human VWF cDNA as a template. Oligonucleotide primers 5′-tga ttc agc gga tgg gcc agg aca gc-3′ and 5′-gct gtc ctg gcc cat ccg ctg aat ca -3′ were used to make the plasmid expressing the mutant VWF with D1529V1530 deletion (ΔD1529V1530). In this study, the amino acid numbering was based on human pro-VWF. The entire cDNA insert for the mutant VWF in the expression vector was verified by sequencing.

### Expression of recombinant VWF

HEK293 cells [34] were cultured in Dulbecco's modified Eagle's medium (DMEM) supplemented with 10% fetal bovine serum at 37°C in an incubator with 5% CO_2_. Transient transfection was performed using LipofectAMINE 2000 (Invitrogen), as described previously [35]. The conditioned medium was collected 48 h after transfection and centrifuged at 15,000 rpm to remove cell debris. The cells were lysed in a buffer containing 100 mM Tris-HCl, pH 7.5, and 1% Triton X-100. Recombinant VWF in the conditioned medium and cell lysates was quantified by ELISA and analyzed for its multimeric structure.

### Confocal immunofluorescence microscopy

For immunofluorescence staining of recombinant VWF in cells [16], HEK293 cells were cultured on coverslips in 6-well plates and transfected with VWF expressing plasmids. After 48 h, the cells were fixed with 4% paraformaldehyde, permeabilized in 1% Triton X-100 and blocked with 5% bovine serum albumin in phosphate-buffered saline. Cells were incubated with first antibodies and then with fluorescence-conjugated secondary antibodies. A polyclonal anti-VWF antibody (Dako) and a monoclonal anti-human PDI antibody (BD Bioscience) were used to visualize VWF and the ER, respectively. Alexa 488- and Alexa 594-conjugated secondary antibodies were from Invitrogen. Fluorescent images were acquired with a confocal microscope (NIKON TE2000-E).
